# History of pelvic fracture management: a review

**DOI:** 10.1186/s13017-016-0075-4

**Published:** 2016-05-04

**Authors:** Philip F. Stahel, E. Mark Hammerberg

**Affiliations:** Department of Orthopaedics, Univesity of Colorado, School of Medicine, Denver Health Medical Center, 777 Bannock Street, Denver, CO 80204 USA; Department of Neurosurgery, Univesity of Colorado, School of Medicine, Denver Health Medical Center, 777 Bannock Street, Denver, CO 80204 USA

**Keywords:** Pelvic fracture, History, Management strategies, Retroperitoneal bleeding, Damage control

## Abstract

High-energy pelvic fractures represent potentially life-threatening injuries due to the risk of acute exsanguinating retroperitoneal hemorrhage. The first report of a severe pelvic ring disruption dates back to Charles Hewitt Moore’s seminal publication from 1851. Significant advantages in the understanding of injury mechanisms and treatment concepts of pelvic ring injuries evolved in the 20^th^ century, and provided the basis to current classification-guided treatment and life-saving “damage control” concepts. However, there is a paucity of reports in the current literature focused on the historic background on the treatment of pelvic ring injuries. The present review was designed to summarize the history and evolution of our current understanding of the mechanisms and management strategies for severe pelvic ring injuries (excluding acetabular fractures which represent a different entity outside of the scope of this article).

## Background

**Fig. 1:**
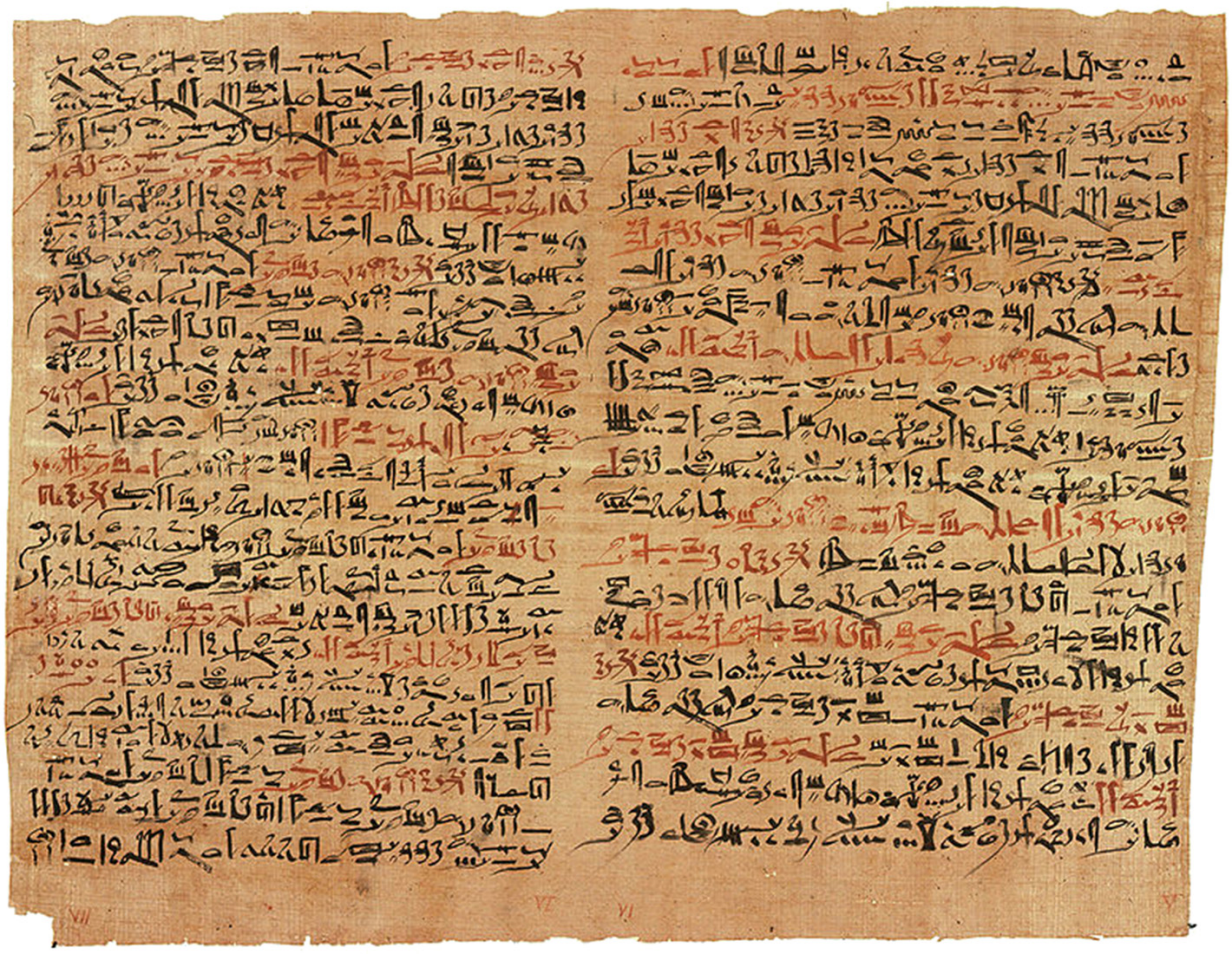
The ‘Edwin Smith Papyrus’ – the oldest documented surgical text in history

The concept of fracture stabilization for pain control, hemostasis, reduction of deformity and fracture healing dates back about 5,000 years to the ancient Egyptians who splinted fractures with wooden sticks and roller bandages [1]. The oldest documented surgical text in history is represented by the “Edwin Smith Papyrus” which dates back to the Old Kingdom in ancient Egypt, around 3,000–2,500 BC (Fig. 1). The papyrus is named after an American Egyptologist who purchased it in Luxor in 1862, and represents a scroll of 4.68 m in length. The text provides an outline on the diagnosis, management principles, and expected outcome of 48 different surgical conditions, including soft tissue injuries, fractures, joint dislocations, and tumors. The management of pelvic fractures is not specifically mentioned in the Edwin Smith Papyrus.

## The ‘Malgaigne era’ (19^th^ century)

**Fig. 2:**
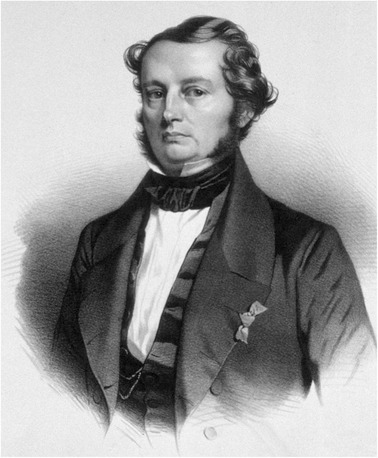
Joseph-Franҫois Malgaigne (1806–1865)

**Fig. 3:**
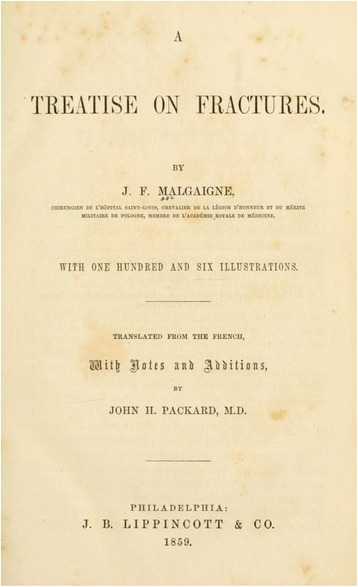
The English translation of Malgaigne’s landmark textbook *‘A treatise on fractures’* (1859)

The modern history of pelvic fracture management begins with the seminal work of Joseph-Franҫois Malgaigne (1806–1865), a French surgeon and world-renowned medical historian (Fig. 2). Malgaigne published multiple influential textbooks on the management strategies of fractures and dislocations, including *“Manuel de médicine opératoire fondée sur l’anatomie normale at l’anatomie pathologique”* (1834), and *“Traité des fractures et des luxations”* (1847), which was published in English translation (‘A treatise on fractures’) in 1859 (Fig. 3). Multiple medical eponyms are associated with the French pioneer, including ‘Malgaigne’s amputation’ (subastragalar amputation with conservation of the talus/astragalus), ‘Malgaigne’s hernia’ (infantile inguinal hernia), ‘Malgaigne’s luxation’ (radial head dislocation/‘nursemaid’s elbow’), and ‘Malgaigne’s fracture’. The latter is the first historic description of a “vertical shear” pelvic ring injury with bilateral sacro-iliac joint dislocations and associated anterior fractures of the pubic rami [2]. Several of these patients sustained injuries after falling or jumping from heights, while others were crushed or run over by horse-drawn carriages. Malgaigne described the resulting injury as a “double fracture” of the anterior and posterior pelvic ring, with displacement of the hemipelvis and shortening of the affected extremity, a case report which was recently made available in English translation [3].

In the absence of radiographic imaging, accurate diagnosis in the 19^th^ century was established by physical exam. Malgaigne described palpation and manipulation to reveal crepitation at the fracture site, and careful estimation of the height of the iliac crest to help rule out a more common fracture pattern associated with lower extremity shortening, i.e. a fracture of the femoral neck. Malgaigne noted that vertically displaced pelvic fractures were often accompanied by impairment or complete loss of lower extremity function. The ‘key’ to successful management of these injuries was the restoration and maintenance of lower extremity length. To this end, Malgaigne advocated an accurate closed reduction maneuver, aided by vaginal and/or rectal palpation. The reduction maneuver was followed by maintenance in a modified traction bed, with application of a pelvic sling, for a minimum of 45 to 50 days. As many patients could not tolerate the prolonged immobilization in traction, most fractures healed with significant limb shortening. Malgaigne noted that many patients would not survive this severe injury, and understood that there was a significant association between this fracture pattern, bleeding, and visceral injuries. The more fortunate patients who survived the initial injury remained at significant risk of delayed suppuration and sepsis, which was likely the result of contaminated open fracture wounds and associated visceral injuries. Therefore, those patients who survived had a grim prognosis in general. As Malgaigne observed: *“If life is preserved, lameness is very apt to ensue.”*

## Charles Hewitt Moore’s case report (1851)

**Fig. 4:**
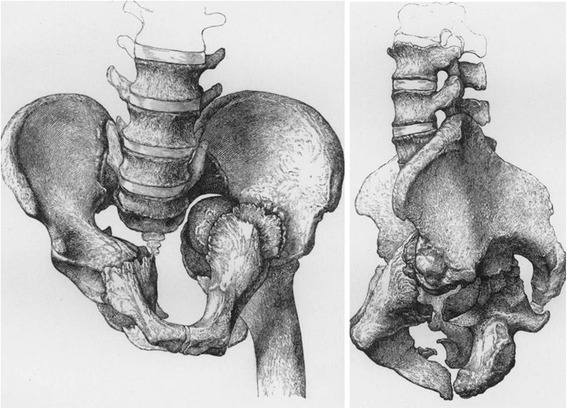
Original graphic artwork from Charles Hewitt Moore’s historic article *‘An account of a case of fracture and distortion of the pelvis, combined with an unusual form of dislocation of the femur’* (1851)

In parallel to Malgaigne’s seminal work in Paris, France, a British surgeon named Charles Hewitt Moore (1821–1870) conducted similar research on pelvic ring disruptions in Plymouth, England [4]. Characterized as a modest person who would *“never speak unless he had something of value to say”* [5], much of Charles Hewitt Moore’s work never met the public eye. A rare case report published in 1851 describes the deforming forces of a severe pelvic ring injury associated with a femoral head protrusion through an acetabular fracture (central hip dislocation) [6]. The injury pattern described in the case report by Dr. Moore represented a rare entity in the 19^th^ century since most patients would either sustain minor pelvic fractures, or succumbed rapidly after major trauma – typically falls from heights – due to associated visceral injuries and acute exsanguination. Moore described the pelvic fracture pattern in excruciating scientific detail, and he emphasized the rare nature of multiple vectors of impacting and deforming forces (Fig. 4): *“Examples are exceedingly rare, however, in which more than one cause of deformity exists in the same pelvis, and there is, I believe, no instance in which so many of the principles of deformity are illustrated as in the accompanying specimen (…)”* [6].

## The ‘Holdsworth era’ (early 20^th^ century)

**Fig. 5:**
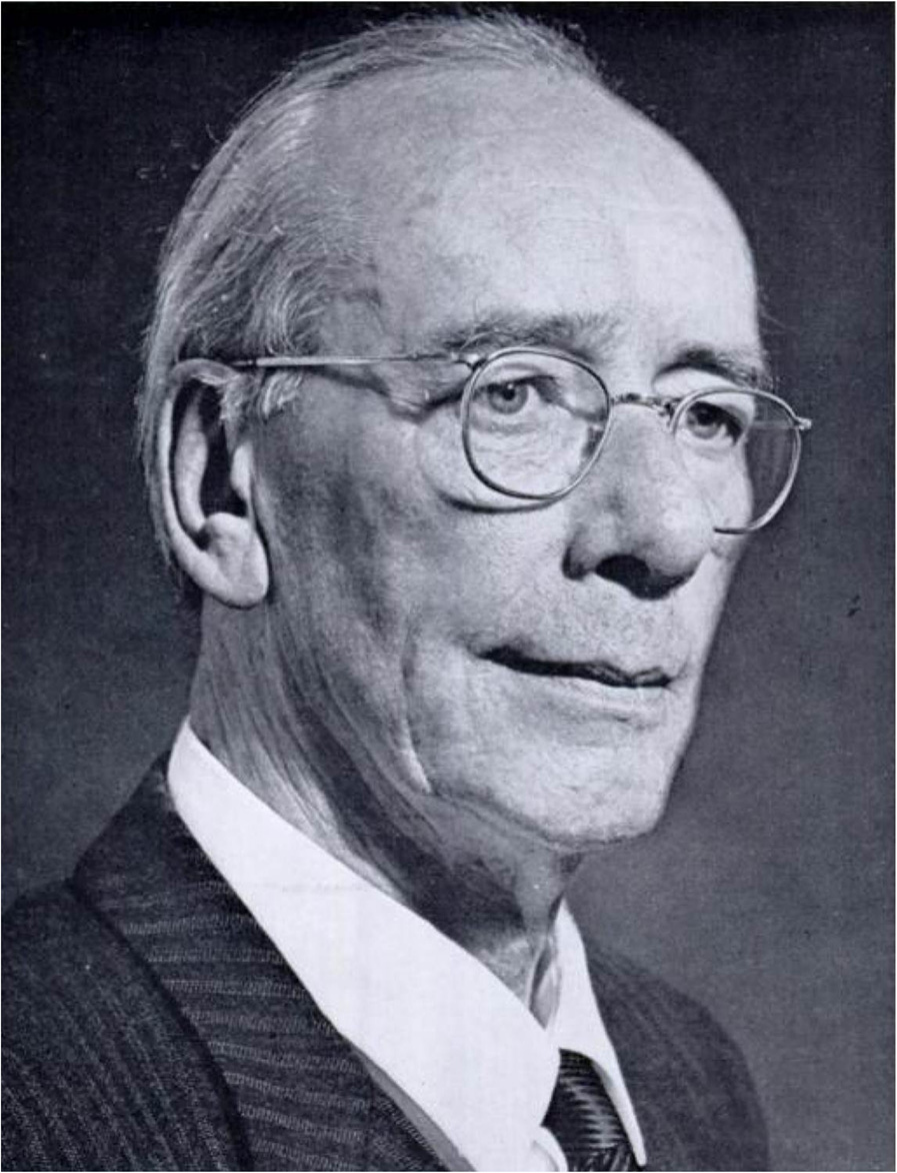
Sir Frank Wild Holdsworth (1904–1969)

**Fig. 6:**
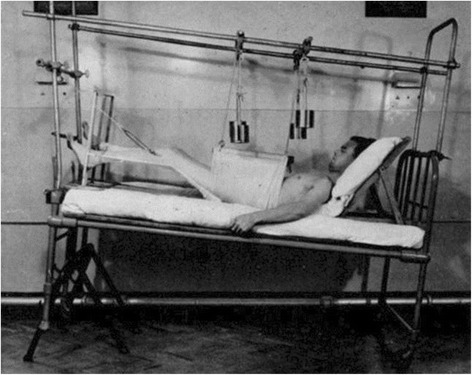
Original photograph depicting the concept of skeletal traction for treatment of a pelvic ring injury in Holdsworth’s original publication (1948)

During the first half of the 20^th^ century, the treatment protocols for pelvic ring injuries remained in line with Malgaigne’s general concept. The introduction of X-ray technology in 1895 by the German physicist Wilhelm Conrad Röntgen (1845–1923) dramatically improved the diagnostic accuracy and classification of these injuries, and allowed to monitor the healing process. Yet, the hallmark of treatment of pelvic ring injuries continued to consist of non-operative management with closed fracture reduction and prolonged bed rest. The application of a pelvic sling with skeletal traction was further refined by Sir Frank Wild Holdsworth (1904–1969), a Professor of Orthopaedics in Yorkshire, England (Fig. 5). Holdsworth’s legacy is mainly funded on the first spine fracture classification, however, he also dedicated significant work to refining the diagnostic and therapeutic strategies for pelvic fractures [7]. In a landmark article from 1948, Holdsworth reported his study of 50 patients with traumatic pelvic ring disruptions during the years 1937–1946 [8]. He described two distinct entities of pelvic ring disruptions, as such: *“1) dislocation of the sacro-iliac joint; 2) fracture of the ilium or sacrum adjacent to the sacro-iliac joint. In both types, there is separation of the symphysis pubis, or fracture of both pubic rami. In both varieties there is displacement of one-half of the pelvis outwards, or outwards and upwards.”* [8]. Holdsworth’s detailed observation reflected the “open book” pattern, the “crescent” lateral compression pattern, and the “vertical shear” injury. He furthermore provided technical recommendations for fracture reduction and immobilization, citing previous seminal work by Sir Astley Cooper [9], Sir Reginald Watson-Jones [10], and Lorenz Böhler [11]. The concept of applying a sling and skeletal traction for treatment of pelvic ring injuries is illustrated in a historic photograph from Holdsworth’s original publication [8] (Fig. 6). Patients were maintained recumbent in a pelvic sling for 12 weeks. Using return to previous employment as a marker for functional recovery, Holdsworth noted that pure sacro-iliac dislocations had a poor prognosis, as fewer than half of these patients returned to their previous work [8]. On the other hand, nearly all of the patients with injuries involving a fracture of the ilium or sacrum returned to heavy labor. Thus, Holdsworth’s landmark article provided a first ‘crude’ classification of pelvic ring injuries, predictive of post-injury outcomes [8].

## Classification-guided management (20^th^/21^st^ century)

The first clinically relevant systematic classification of pelvic fractures, based on the mechanism of injury, was described by Pennal and Sutherland in 1961 [12]. This system defines three distinct categories of pelvic ring injuries: (1) avulsion fractures, (2) ‘stable’ fractures, and (3) ‘unstable’ fractures, and attempts to correlate injury severity with outcomes. Dunn and Morris later revisited the non-operative concept for the management of pelvic ring injuries and dislocations, based on the Pennal/Sutherland classification system [13].

In 1980, Pennal and Tile introduced the aspect of fracture stability to the original Pennal/Sutherland classification and incorporated mechanisms and vectors of injury [14]. The Pennal/Tile classification furthermore served as a basis for therapeutic decision-making and management protocols of pelvic ring injurie [15]. Currently used classification systems are largely based on the seminal publications by Tile, Pennal, and Sutherland. For example, the AO/OTA classification for pelvic ring injuries [16] is mainly based on Marvin Tile’s original classification system from 1980, and the classification by Young & Burgess [17] is based on the original Pennal/Sutherland description from 1961. Both the Tile and Young & Burgess classification systems are still widely used in the 21^st^ century for decision-making and guidance of therapeutic protocols in the acute management of patients with pelvic ring disruptions [18, 19].

By the middle part of the 20^th^ century, with a growing number of high-speed motor vehicle accidents, it had become clear that pelvic fractures were involved in a significant number of fatal injuries, mostly related to exsanguinating retroperitoneal hemorrhage [20]. At this time, resuscitation strategies were in their infancy, and there was ongoing debate regarding the appropriate sequence and surgical priorities in the acute management of pelvic hemorrhage [21]. The role of the orthopaedic surgeon in the acute management and resuscitation of patients with pelvic ring disruptions continued to grow in the second half of the 20^th^ century. External fixation of pelvic ring injuries was introduced and applied increasingly in the early management of hemodynamically unstable patients [22–24]. The underlying theory was that external fixation might decrease ongoing blood loss by eliminating motion at the fracture site. In addition, by reducing ‘open book’ injuries, external fixation was thought to reduce the intrapelvic volume and to help reducing retroperitoneal blood loss [25].

While there are some early reports from the 1950s on internal fixation for acute pelvic fractures [26], the majority of pelvic ring injuries were managed non-operatively at the time. During the second half of the 20^th^ century, treatment protocols moved beyond conservative treatment strategies, as a number of surgeons began to recommend surgical fixation for selected pelvic ring injuries. Marvin Tile was a pioneer in this field and he used his own classification system to guide treatment recommendations [25]. Initially, definitive internal fixation was reserved for vertically unstable fractures [25]. However, into the 1980s, indications for definitive internal fixation were broadened to include rotationally unstable fractures as well [27–29]. The notion that surgical fixation of unstable pelvic ring injuries allows early mobilization of patients and provides superior clinical outcomes became prevalent towards the end of the 20^th^ century in North American and European countries [30, 31]. This experience solidified the concept of early internal fixation of unstable pelvic ring injuries as a new international standard of care in the 21^st^ century.

## Conclusions

Significant progress has been made in recent years in the acute management of severe pelvic ring disruptions by mitigating the risk of acute exsanguinating hemorrhage and associated post-injury mortality. The historic evolution related to our understanding of underlying injury mechanisms has provided the basis for classification-guided management strategies for high-energy pelvic injuries. Future innovations on the horizon include less-invasive management strategies, e.g. by early definitive care with percutaneous fixation of unstable pelvic ring disruptions, and bedside point-of-care resuscitation of hemorrhagic shock and post-injury coagulopathy which represents the current “frontier” of cutting-edge research in the 21^st^ century [32–38].
